# Supplementary material for: Inhibition of PRMT5/MEP50 Arginine Methyltransferase Activity Causes Cancer Vulnerability in NDRG2low Adult T-Cell Leukemia/Lymphoma
Source: Int J Mol Sci. 2024 Feb 29;25(5):2842. doi: 10.3390/ijms25052842 (PMC10932150; doi:10.3390/ijms25052842)
Supplement: Supplementary file 1 [file ijms-25-02842-s001.zip › ijms-2865653-supplementary.pdf]

Supplementary Table S1. The list of the antibodies used in this manuscript.

Antibody	Manufacturer	Catalog no	Type	Dilution
AKT	Cell Signaling	#9272	Rabbit polyclonal	1/1000
MEP50	Cell Signaling	#2823	Rabbit polyclonal	1/1000
H3R8me2s	abcam	ab130740	Rabbit polyclonal	1/1000
H4R3me2s	abcam	ab5823	Rabbit polyclonal	1/1000
HSP90α/β(F-8)	SANTA CRUZ	sc-13119	Mouse monoclonal	1/1000
NDRG2(E-20)	SANTA CRUZ	sc-19468	Goat polyclonal	1/1000
PRMT5(A-11)	SANTA CRUZ	sc-376937	Mouse monoclonal	1/1000
NEMO(F-10)	SANTA CRUZ	sc-166398	Mouse monoclonal	1/1000
GFP(B-2)	SANTA CRUZ	sc-9996	Mouse monoclonal	1/1000
SYM10	Millipore	07-412	Rabbit antiserum	1/1000
β-actin(AC-74)	Sigma-Aldrich	A5316	Mouse monoclonal	1/1000
Flag(M2)	Sigma-Aldrich	F3165	Mouse monoclonal	1/1000
HA(3F10)	Roche	11867423001	Rat monoclonal	1/1000
Polyclonal Rabbit anti-Mouse IgG/HPR	Dako	P0260		1/1000
Polyclonal Swine anti-Rabbit IgG/HPR	Dako	P0399		1/1000
Polyclonal Rabbit anti-Rat IgG/HPR	Dako	P0450		1/1000
Polyclonal Rabbit anti-Goat IgG/HPR	Dako	P0449		1/1000

Antibodies were from Cell Signaling Technologies (Beverly, MA, USA), abcam (Boston, MA, USA), Santa Cruz Biotechnology (Santa Cruz, CA, USA), Millipore (Bedford, MA, USA), Sigma-Aldrich (St. Louis, MO, USA), Roche (Basel, Switzerland), and DAKO (Carpinteria, CA, USA).

Supplementary Table S2. The primer sequences to construct expression vectors with shRNA sequences.

Name		Sequence (5' to 3')
shMEP50-1	sense	CTGTCAGTGGTAGCAAAGA
	antisense	TCTTTGCTACCACTGACAG
shMEP50-2	sense	CACCAAGAGTACAAGCTGT
	anti-ense	ACAGCTTGTACTCTTGGTG
shPRMT5	sense	CCGAAATAGCTGACACACT
(mouse)	antisense	AGTGTGTCAGCTATTTCGG

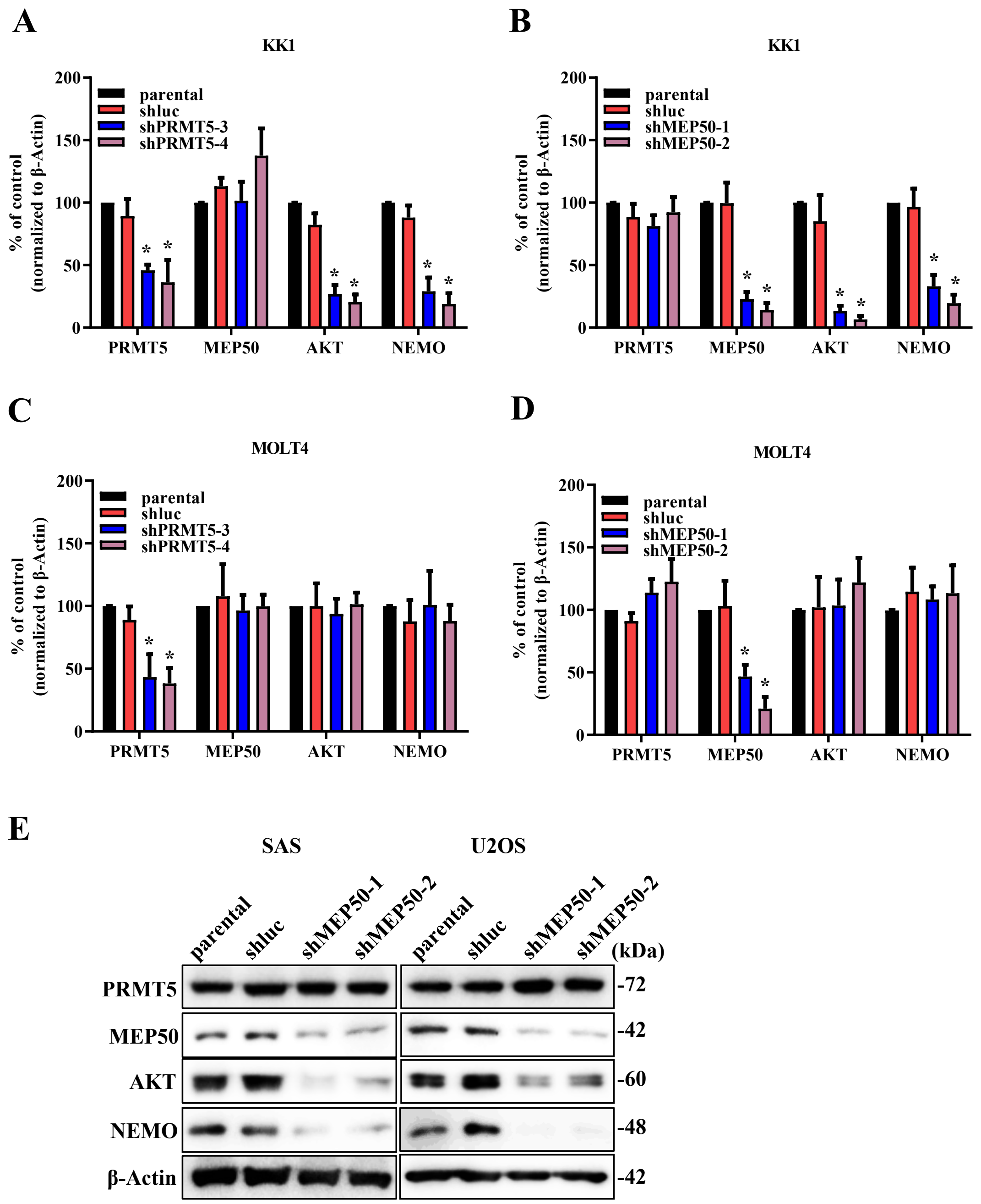

Supplementary Figure S1. The knockdown of PRMT5/MEP50 expression results in the inhibition of cell proliferation through the degradation of client proteins in ATL and various cancer cells with low NDRG2 expression.

(A) Expression of PRMT5, MEP50, AKT, and NEMO in KK1 cells (parental, shluc, shPRMT5-3, and 4) was immunoblotted by each specific antibody. Bar graphs show the quantification of the relative band intensity normalised to β-actin. The mean and s.d. are shown (n=3), with * p<0.05, compared with parental.

(B) Expression of PRMT5, MEP50, AKT, and NEMO in KK1 cells (parental, shluc, shMEP50-1, and 2) was immunoblotted by each specific antibody. Bar graphs show the quantification of the relative band intensity normalised to β-actin. The mean and s.d. are shown (n=3), with * p<0.05, compared with parental.

(C) Expression of PRMT5, MEP50, AKT, and NEMO in MOLT4 cells (parental, shluc, shPRMT5-3, and 4) was immunoblotted by each specific antibody. Bar graphs show the quantification of the relative band intensity normalised to β-actin. The mean and s.d. are shown (n=3), with * p<0.05, compared with parental.

(D) Expression of PRMT5, MEP50, AKT, and NEMO in MOLT4 cells (parental, shluc, shMEP50-1, and 2) were immunoblotted by each specific antibody. Bar graphs show the quantification of the relative band intensity normalised to β-actin. The mean and s.d. are shown (n=3), with * p<0.05, compared with parental.

(E) Expression of PRMT5, MEP50, AKT, and NEMO in SAS and U2OS cells (parental, shluc, shMEP50-1, and 2) was immunoblotted by each specific antibody.

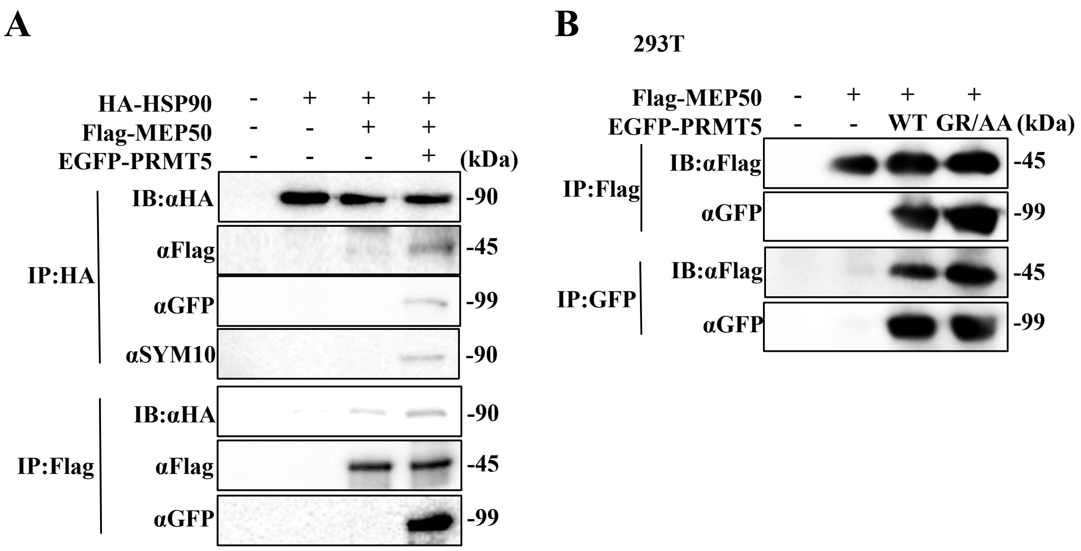

Supplementary Figure S2. Hyperphosphorylated PRMT5 binds to MEP50 and promotes HSP90 arginine methylation.

(A) The lysates of 293T cells transfected with HA-tagged HSP90, Flag-tagged MEP50, and/or EGFP-tagged PRMT5 were immunoprecipitated with anti-HA or anti-Flag antibody, and immunoprecipitates were assayed by western blotting with the indicated antibodies.

(B) EGFP-tagged wild-type or mutated PRMT5 (GR/AA) was co-transfected with Flag-tagged MEP50. The cell lysates were immunoprecipitated with anti-Flag antibody for MEP50 or anti-GFP antibody for PRMT5 and then immunoblotted by each indicated antibody.

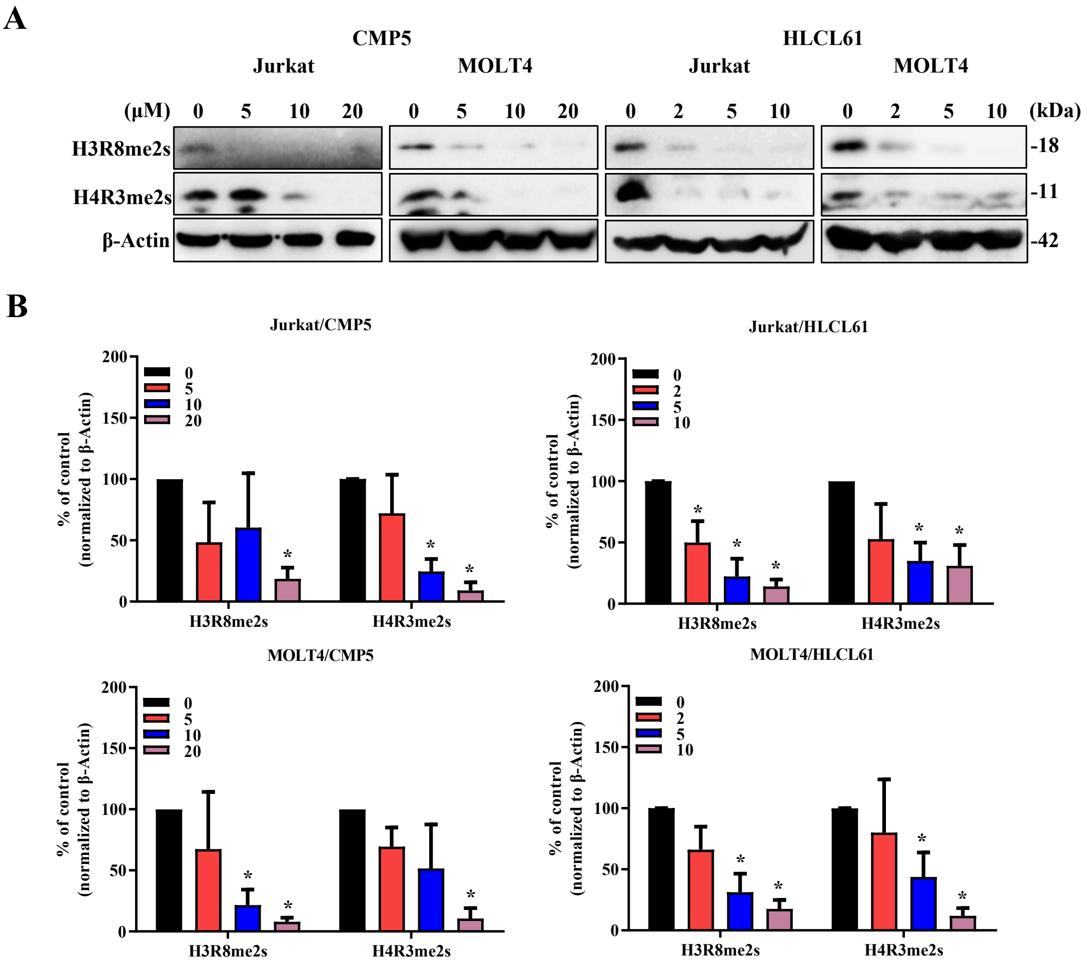

Supplementary Figure S3. NDRG2^{low} ATL cells are sensitive to PRMT5 inhibitors.

(A) Expression of H3R8me2s and H4R3me2s in Jurkat and MOLT4 cells after treatment with the indicated doses of CMP5 and HLCL61 for 24 h were determined via immunoblot analysis.

(B) Bar graphs show the quantification of the relative band intensity normalised to β-actin. The mean and s.d. are shown (n=3), with * $p<0.05$, compared with 0.

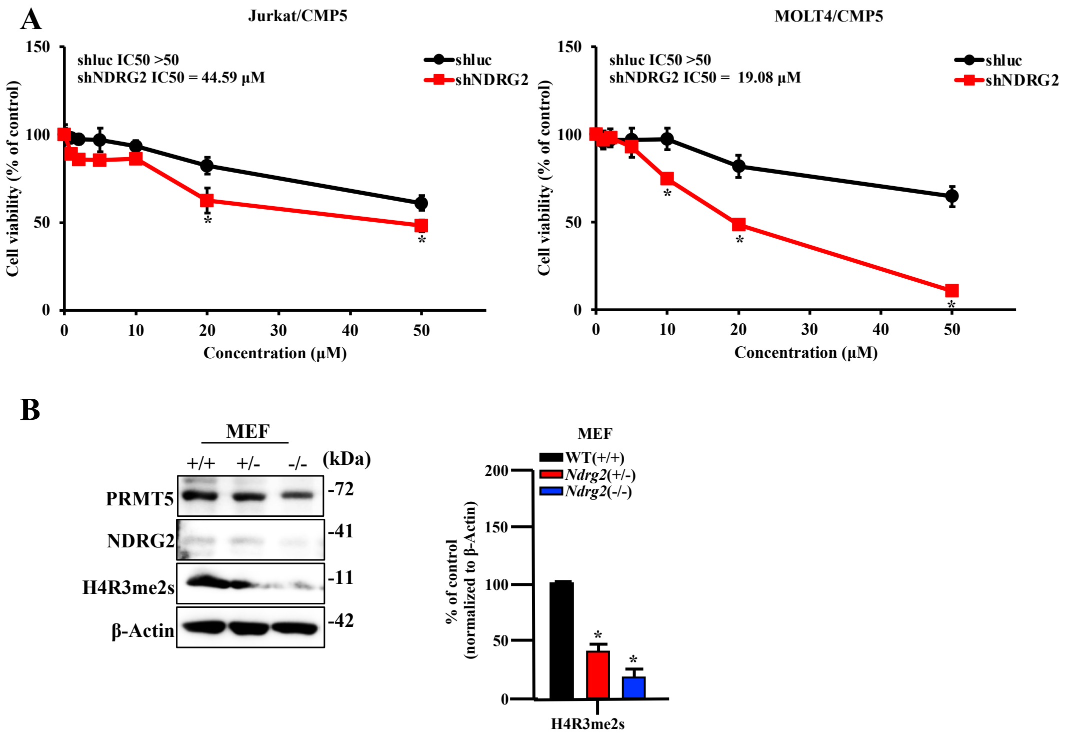

Supplementary Figure S4. Knockdown of NDRG2 expression in T-ALL cells enhances sensitivity to PRMT5 inhibitors.

(A) Cell viability and IC50 were determined after treatment with 0-50 μM CMP5 at 120 h in Jurkat and MOLT4 cells (shluc and shNDRG2). The mean and s.d. are shown (n=4), with *: p<0.05 compared with shluc.

(B) Expression of PRMT5, NDRG2, and H4R3me2s in WT(+/+), *Ndrg2*(+/-), and *Ndrg2*(-/-) MEFs was immunoblotted by each specific antibody. Bar graphs show the quantification of the relative band intensity normalised to β-actin. The mean and s.d. are shown (n=3), with * p<0.05, compared with WT(+/+).

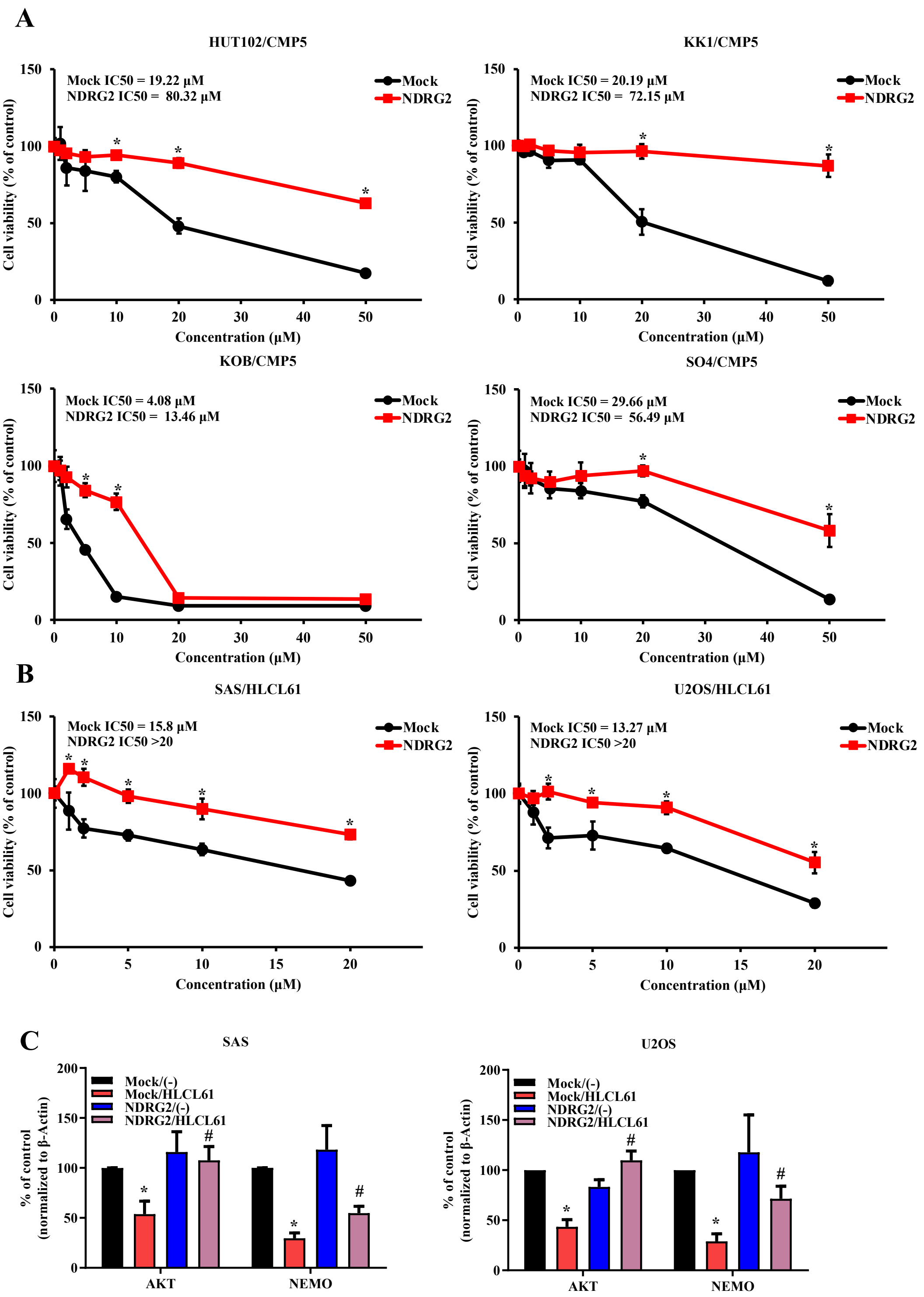

Supplementary Figure S5. The enhanced expression of NDRG2 attenuates the antitumour effects of PRMT5 inhibitors in ATL and solid cancer cells.

(A) Cell viability and IC50 were determined after treatment with 0-50 μM CMP5 at 120 h in HUT102, KOB, KK1, and SO4 cells (Mock and NDRG2). The mean and s.d. are shown (n=4), with *: $p<0.05$ compared with Mock.

(D) Cell viability and IC50 were determined after treatment with 0-20 μM HLCL61 at 120 h in SAS and U2OS cells (Mock and NDRG2). The mean and s.d. are shown (n=4), with *: $p<0.05$ compared with Mock/(-) and # $p<0.05$ compared with Mock/HLCL61.

(E) Expression of AKT and NEMO in SAS and U2OS cells (Mock and NDRG2) after treatment with 10 μM HLCL61 for 24 h was determined via immunoblot analysis. Bar graphs show the quantification of the relative band intensity normalised to β-actin. The mean and s.d. are shown (n=3), with * $p<0.05$, compared with parental.
